# Pan-cancer analysis of *SETD2* mutation and its association with the efficacy of immunotherapy

**DOI:** 10.1038/s41698-021-00193-0

**Published:** 2021-06-14

**Authors:** Mingdong Lu, Bin Zhao, Mengshan Liu, Le Wu, Yingying Li, Yingna Zhai, Xian Shen

**Affiliations:** The Second Affiliated Hospital and Yuying Children’s Hospital of Wenzhou Medical University, Wenzhou, China

**Keywords:** Tumour biomarkers, Predictive markers

## Abstract

Histone methyltransferase SETD2 plays a critical role in maintaining genomic integrity and stability. Here, we investigated the characteristics of *SETD2* somatic mutation in the cancer genome atlas pan-cancer cohort. Our data revealed that, compared with *SETD2* nonmutant patients, *SETD2* mutant patients had higher tumor mutation burden and microsatellite instability. In addition, the transcriptions of most genes related to immune activities were upregulated in patients with *SETD2* mutant tumors. Further examination of cancer patients treated with immune checkpoint inhibitors suggested *SETD2* mutation was associated with favorable clinical outcomes. These results have implication for the personalization of cancer immunotherapy.

Immune checkpoint inhibitors (ICIs) targeting programmed cell death protein-1 (PD-1), programmed cell death ligand 1 (PD-L1), and cytotoxic T-lymphocyte-associated antigen 4 (CTLA-4) can significantly improve the overall survival (OS) in cancer patients^1^. However, most patients cannot benefit from immunotherapy and reliable biomarkers are warranted^2^. Although the US Food and Drug Administration (FDA) has approved the application of PD-L1, defective mismatch repair or microsatellite instability high (dMMR/MSI-H), and tumor mutation burden (TMB) in clinical practice, we and others have shown these biomarkers are imperfect^2,3^.

Histone methyltransferase SETD2, the sole human gene responsible for the trimethylation of histone H3 at lysine 36 (H3K36me3), plays a critical role in maintaining genomic integrity and stability by several distinct pathways^4^. Pfister et al. found SETD2 was necessary for homologous recombination repair^5^, depletion of *SETD2* shows MSI and an increased spontaneous mutation frequency, characteristic of dMMR cells^6^. SETD2 also provides an alternative mechanism that leads to DNA damage repair through interacting with p53 tumor suppressor^7^. Moreover, SETD2 can directly change the chromatin accessibility, which will generate RNA processing defects^8^. It is estimated that mRNA processing defects occur in 25% of expressed gene across the whole genome when *SETD2* is mutant^8^. We speculate the mutation of *SETD2* results in the enrichment of tumor mutation-specific neo-antigens in the cell surface, the immune system will recognize and attack these cells with the help of ICIs. The unique features of *SETD2* mutation makes it a potential biomarker for cancer immunotherapy. Accordingly, with accumulated data that are publicly available, here we conducted a comprehensive analysis to examine the characteristics of *SETD2* mutation and its association with the efficacy of immunotherapy.

We first examined the prevalence of *SETD2* somatic mutations in the cancer genome atlas (TCGA) pan-cancer cohort. Of all 10,427 patients, 451 (4.33%) harbored *SETD2* mutations (Fig. 1a). *SETD2* mutations occurred in a small subset of most tumor types, and the mutant frequencies differed significantly across various tumors (*P* < 0.001). Totally, 569 *SETD2* mutations were identified, 375 (65.9%) were missense mutations, 193 (33.9%) were truncating mutations, and 1 (0.2%) was inframe mutation (Fig. 1b). These mutations occurred in a dispersed manner throughout the whole sequence (Fig. 1b) and 3D protein structure (Fig. 1c).

**Fig. 1:**
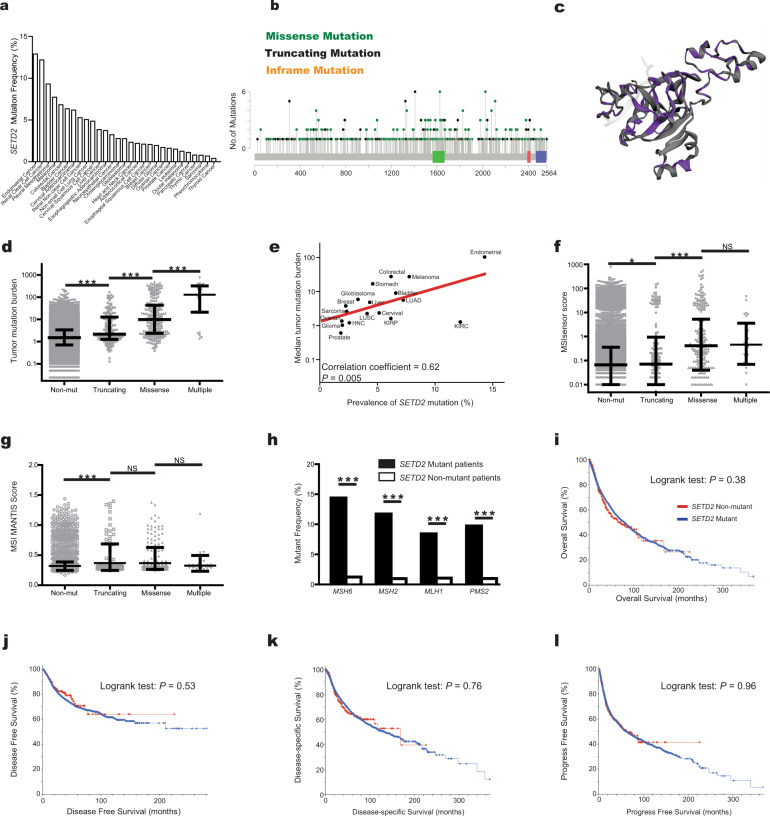
The characteristics of *SETD2* mutations in TCGA pan-cancer cohort. **a** The prevalence of *SETD2* mutations across tumors. **b** The subtypes and distributions of *SETD2* somatic mutations. *X*-axis, amino acid; *Y*-axis, numbers of *SETD2* mutations; green box, SET domain (1561–1667); red box, WW domain (2391–2420); blue box, SRI domain (2466–2558); green dot, missense mutation; black dot, truncating mutation; orange dot, inframe mutation. **c** Location of variants on the 3D protein structure of SETD2. Purple, mutated amino acid. **d** Tumor mutation burden (TMB) in *SETD2* nonmutant cancer and different subtypes of *SETD2* mutant cancer. Each gray dot represents one patient, black line represents the median TMB and its interquartile ranges. **e** The prevalence of *SETD2* mutation and median TMB in multiple tumors. Red line, fitted curve; HNC, head and neck cancer; LUAD, lung adenocarcinoma; LUSC, lung squamous cell carcinoma; KIRC, kidney renal clear cell carcinoma; KIRP, kidney renal papillary cell carcinoma. **f** MSIsensor scores in *SETD2* nonmutant cancer and different subtypes of *SETD2* mutant cancer. **g** MSI MANTIS scores in *SETD2* nonmutant cancer and different subtypes of *SETD2* mutant cancer. **h** The mutant frequencies of *MSH2, MSH6*, *MLH1*, and *PMS2* in *SETD2* mutant and nonmutant cancer. **i** Overall survival (OS) analysis stratified by *SETD2* mutation status in the whole TCGA cohort. **j** Disease-free survival (DFS) analysis stratified by *SETD2* mutation status in TCGA. **k** Disease-specific survival (DSS) analysis stratified by *SETD2* mutation status in TCGA. **l** Progress-free survival (PFS) analysis stratified by *SETD2* mutation status in TCGA. NS, *P* > 0.05; *P < 0.05; ***P < 0.001.

**Fig. 2:**
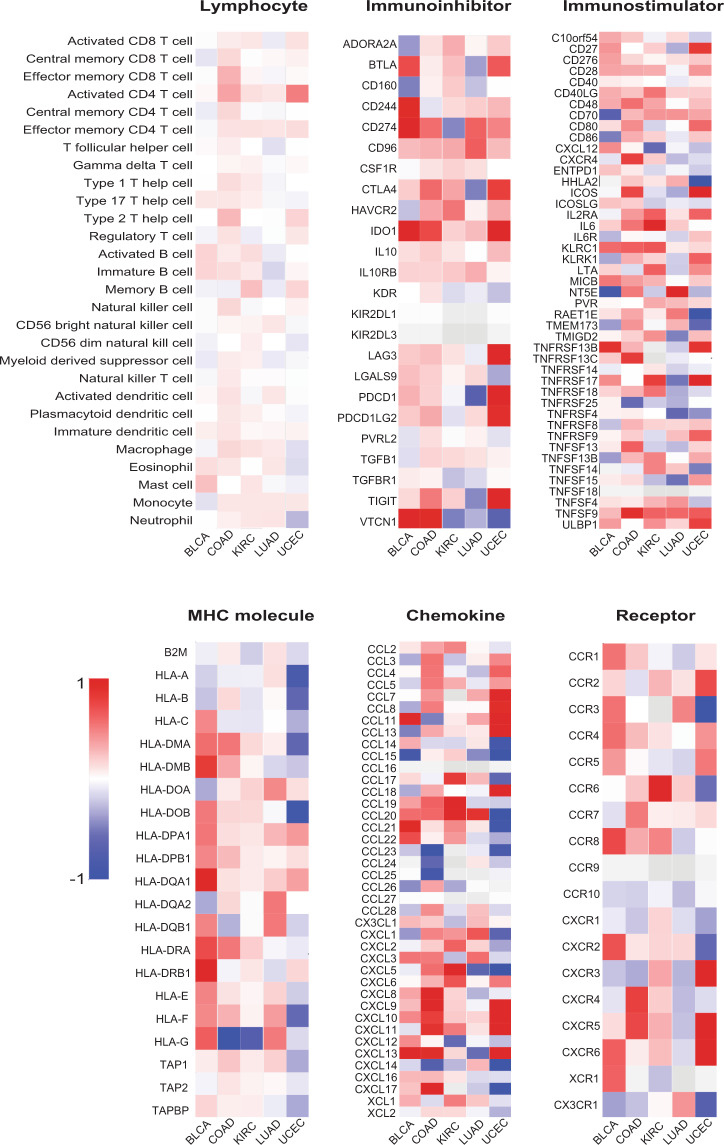
*SETD2* mutation and immune features in cancer. The differences of median gene expression between *SETD2* mutant samples and *SETD2* nonmutant samples in bladder urothelial carcinoma (BLCA), colon adenocarcinoma (COAD), kidney renal clear cell carcinoma (KIRC), lung adenocarcinoma (LUAD), and uterine corpus endometrial carcinoma (UCEC).

In TCGA cohort, higher TMB was observed in patients with *SETD2* mutant cancer (median, 5.9; interquartile range, 1.8–29.2) than those in patients with *SETD2* nonmutant disease (1.5, 0.7–3.3; *P* < 0.0001). Moreover, TMB was significant different among *SETD2* truncating mutant cancer (2.2,1.–10.2), *SETD2* missense mutant cancer (9.4, 2.3–39.5), and cancer with multiple mutations (118.1, 21.2–270.7; Fig. 1d). TMB stratified by *SETD2* mutation status in different tumors were presented in Supplemental Fig. 1a. In colorectal cancer, further analysis revealed that TMB in non-MSI *SETD2* mutant tumors (4.9, 2.8–132.2) was significantly higher than TMB in non-MSI *SETD2* nonmutant tumors (2.5, 2.0–3.3; P < 0.0001). Interestingly, we observed a significant correlation between the frequencies of *SETD2* mutation and median tumor mutation burdens across multiple tumor types (correlation coefficient, 0.62; *P* = 0.005; Fig. 1e).

MSIsensor is an effective and efficient tool for deriving MSI status^9^. MSIsensor scores in patients with *SETD2* mutant cancer (0.12; 0.01–0.84) were significantly higher than the scores in patient with *SETD2* nonmutant cancer (0.05, 0.00–0.31; *P* < 0.0001; Fig. 1f). There was no correlation between the frequency of *SETD2* mutation and median MSIsensor scores (correlation coefficient, 0.10; *P* = 0.71). The associations between MSIsensor scores and *SETD2* mutation in different tumors were presented in Supplemental Fig. 1b. To further validate the association between *SETD2* mutation and MSI status, we also examined the MSI MANTIS^10^ scores in patients with *SETD2* mutant cancer (0.32, 0.30–0.34) and patients with *SETD2* nonmutant cancer (0.31, 0.29–0.33; *P* < 0.0001). Of note, the scores showed no differences among various subtypes of *SETD2* mutation (Fig. 1g). *MSH2, MSH6*, *MLH1*, and *PMS2* played critical roles during the mismatch repair (MMR) process^11,12^, the mutation in any of these four MMR genes might cause MSI-H. Here, we investigated the co-occurrence patterns of these four MMR mutant genes and *SETD2* mutation (Fig. 1h). Compared with patients with *SETD2* nonmutant cancer, patients with *SETD2* mutant cancer harbored more MMR mutant genes (*MSH6*, 1.24% vs.14.38%; *MSH2*, 0.98% vs.11.73%; *MLH1*, 1.06% vs.8.41%; *PMS2*, 0.98% vs.9.73%; *P* < 0.0001 for all four genes).

Next, we investigated the correlations between *SETD2* mutation and various immune signatures, including 28 tumor-infiltrating lymphocytes, 24 immunoinhibitors, 45 immunostimulators, 21 major histocompatibility complex molecules, 40 chemokines, and 18 chemokine receptors, in kidney renal clear cell carcinoma (KIRC, *n* = 43), colon adenocarcinoma (COAD, *n* = 41), lung adenocarcinoma (LUAD, *n* = 30), bladder urothelial carcinoma (BLCA, *n* = 27), and uterine corpus endometrial carcinoma (UCEC, *n* = 22), five tumors with over 20 *SETD2* mutant cases in TCGA cohort (Fig. 2). Compared with *SETD2* nonmutant samples, most immune-related genes were upregulated in *SETD2* mutant samples, and many showed statistically significant. These results suggested the immune system was more active in *SETD2* mutant cancer, which might be recognized as immunologically “hot” tumor. Moreover, our data provided strong evidence that cancer epigenetic driver mutations could shape tumor immune phenotype.

**Fig. 3:**
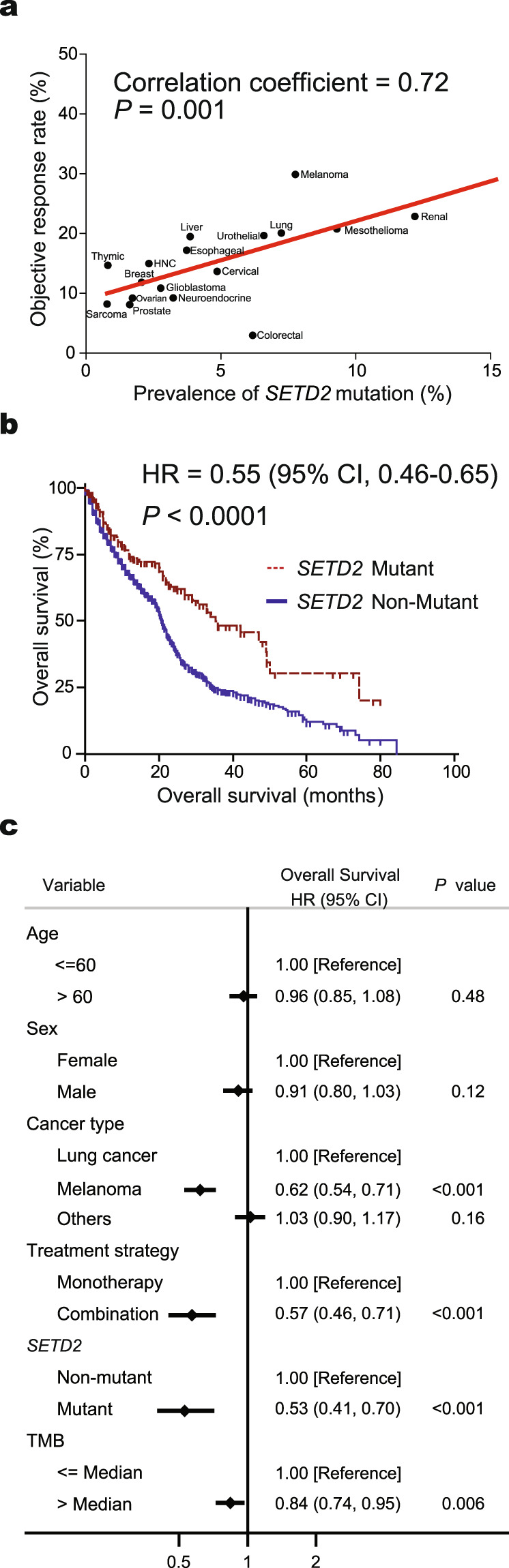
*SETD2* mutation and the efficacy of immunotherapy. **a** The correlation between the frequencies of *SETD2* mutation and objective response rates across multiple tumors. Red line, fitted curve; HNC, head and neck cancer. **b** Kaplan–Meier survival analysis stratified by *SETD2* mutation status in 2734 cancer patients treated with immune checkpoint inhibitors. CI, confidence interval; HR, hazard ratio. **c** Multivariate analysis of the association between *SETD2* mutation and overall survival.

To investigate whether these distinct characterisitics of *SETD2* mutation could translate into cancer prognosis, we compared the OS (*P* = 0.38, Fig. 1i), disease-free survival (*P* = 0.53, Fig. 1j), disease-specific survival (*P* = 0.76, Fig. 1k), and progress-free survival (*P* = 0.96, Fig. 1l) between patients with *SETD2* mutant cancer and patients with *SETD2* nonmutant cancer. The prognosis and survival for cancer patients in TCGA cohort were independent of *SETD2* mutant status.

Previous studies suggested that copy number alteration (CNA) of *SETD2* contributed to the nucleosome stabilization, coordination of DNA repair, and suppression of replication stress^13^. Hence, we examined the features of cancer patients with CNA of *SETD2*. Totally, 75 patients (0.68%) with *SETD2* CNA were identified in TCGA cohort. The frequencies of the *SETD2* CNA acorss different tumors were shown in Supplemental Fig. 2a. The CNA of *SETD2* was not associated with TMB (*P* = 0.73, Supplemental Fig. 2b), MSI MANTIS scores (*P* = 0.32, Supplemental Fig. 2c), MSIsensor scores (*P* = 0.71, Supplemental Fig. 2d), and OS (*P* = 0.63, Supplemental Fig. 2e). It should be noted that, due to the limited number of *SETD2* CNA patients included in the TCGA cohort, further investigations are needed to confirm these results.

Our previous study including 22,165 patients treated with PD-1/PD-L1 blockade monotherapy from 160 trials demonstrated the objective response rates (ORRs) in various tumors^1^. With the frequencies of *SETD2* mutations extracted from TCGA, we found that there was a significant correlation between the prevalences of *SETD2* mutations and ORRs (correlation coefficient, 0.72; *P* = 0.001, Fig. 3a).

For survival analysis, a total of 2734 patients from eight studies were included (Table 1). *SETD2* mutation was associated with significantly better OS (hazard ratio (HR), 0.55; 95% confidence interval (CI), 0.46–0.65; *P* < 0.0001; Fig. 3b). This association remained robust after adjusting for confounding factors, including age, sex, cancer type, treatment strategy, and TMB (Fig. 3c), suggesting *SETD2* mutation was not a prognostic, but a predictive biomarker for cancer immunotherapy.

**Table 1:** Baseline features of 8 eligible studies included in the immunotherapy analysis.

Study	Drugs	Cancer type	*SETD2* mutation status	No. patients	Sex (male/female)	Age(mean, range, year)
Van Allen^17^	Ipilimumab	Melanoma	Positive	10	7/3	63(32–83)
			Negative	100	71/29	58(18–86)
Hugo^18^	Pembrolizumab/nivolumab	Melanoma	Positive	5	2/3	63(27–82)
			Negative	32	24/8	60(19–84)
Riaz^19^	Nivolumab	Melanoma	Positive	2	NA	NA
			Negative	71	NA	NA
Miao^23^	Nivolumab	Renal cancer	Positive	15	11/4	62(50–69)
			Negative	20	11/9	62(36–77)
Miao^24^	Anti-CTLA-4, anti-PD-1, and anti-PD-L1	Microstatellite-stable tumors	Positive	18	11/7	67(39–83)
			Negative	231	143/88	59(18–86)
Samstein^25^	Anti-CTLA-4, anti-PD-1, and anti-PD-L1	Multiple tumors	Positive	131	102/29	63(19–90)
			Negative	1530	932/598	61(15–90)
POPLAR^20,21^	Atezolizumab	Lung cancer	Positive	7	5/2	60(42–74)
			Negative	137	88/49	61(42–82)
OAK^21,22^	Atezolizumab	Lung cancer	Positive	17	10/7	65(39–80)
			Negative	408	251/157	63(33–82)

*NA* not available.

Due to the success of POPLAR and OAK, two multicenter randomized controlled trials conducted in patients with non-small cell lung cancer, FDA granted the application of atezolizumab in clinical practice^14^. Here, we specifically examined the association between *SETD2* mutation and various clinicopathological characteristics in patients enrolled in POPLAR and OAK. As shown in Table 2, more PD-L1-positive tumors and higher TMBs were discovered in patients with *SETD2* mutant cancer.

**Table 2:** The clinicopatholgical characteristics of patients included in POPLAR and OAK trials.

	*SETD2* mutant	*SETD2* nonmutant	*P*
Number of patients	24	545	
Age(median, range, year)	63(39–80)	63(33–82)	0.34
Race (White/Other, %)	83/17	72/28	0.11
Sex (male/female, %)	63/37	62/38	0.49
Smoking status (current/former/never, %)	21/71/8	14/66/20	0.07
ECOG performance status (1/0, %)	67/33	64/36	0.40
Subtype (squamous/non-squamous, %)	38/62	28/72	0.15
Line of treatment (second/third, %)	63/37	73/27	0.13
Mean diameter of target lesion	78.88	77.52	0.45
Mean number of metastatic sites	3.04	2.91	0.33
*KRAS* mutant status (positive/negative/unknown, %)	0/21/79	7/22/71	0.11
*EGFR* mutant status (positive/negative/unknown, %)	8/63/29	9/69/22	0.50
*EML4-ALK* mutant status (positive/negative/unknown, %)	0/63/37	0/49/51	0.37
PD-L1 expression^a^ (positive/negative/unknown, %)	58/13/29	42/32/26	**0.02**
TMB (mean ± SE)	17.13 ± 2.32	10.65 ± 0.45	**0.001**

*ECOG* Eastern Cooperative Oncolgy group.

^a^The threshold for PD-L1 positivity and negativity was that PD-L1 stained cell accounted for 1% of tumor cells or immune cells.

The bold values mean *P* < 0.05.

In summary, our data reveal that *SETD2* mutation is correlated with higher tumor mutation burden and MSI, and more immune activities in cancer. Moreover, *SETD2* mutation status is a potential biomarker in predicting the clinical outcomes in patients treated with ICIs.

## Methods

### Study design

Our study was deemed exempt from institutional board approval and patient informed consent because all data are deidentified and publicly available. The nonsynonymous mutations were defined as frameshift, missense, nonsense, splice site, nonstop, and translation start site changes. Truncating mutations were defined as nonsense, nonstop, frameshift deletion, frameshift insertion, and splice site. Inframe mutations included inframe deletion and inframe insertion.

### TCGA data

TCGA database included sequencing and clinicopathological data from patients with over 30 types of tumors. All data included for prevalence analysis of *SETD2* mutations and CNA, subtype analysis, 3D protein structure, mutation counts, MSIsensor score, MSI MANTIS score, and survival analysis were queried and downloaded from the cBioPortal for Cancer Genomics database (https://www.cbioportal.org)^15^. To study the association between *SETD2* mutation and immune characteristics, KIRC, COAD, LUAD, BLCA, and UCEC data obtained from TCGA were analyzed using TISIDB (http://cis.hku.hk/TISIDB)^16^, a database integrated multiple types of data resources in onco-immunology.

### Data analysis of patients with immunotherapy

We searched “immune checkpoint blockade clinical trials” across all tumor types on ClinicalTrials.gov for status as completed. The treatment strategies were classified as anti-PD-L1 (avelumab, atezolizumab, and durvalumab), anti-PD-1 (nivolumab, pembrolizumab, and cemiplimab), and anti-CTLA-4 (ipilimumab and tremelimumab), in each tumor type. Then, we conducted systematic search of PubMed database for potential trials in November 2020. Two investigators (M.L. and B.Z.) independently screened the full texts were checked for their eligibility. Any discrepancy was resolved by discussion. The selection criteria were prespecified. To be eligible, studies had to meet the following standards: (1) population: clinical trials including over 30 adult patients with solid tumor; (2) intervention: at least one arm in the trial was treated with ICIs irrespective the dosage and duration of the treatment; and (3) outcomes: reported information regarding *SETD2* mutation status and OS. In addition, the reference lists of all trials fulfilling the eligibility criteria were also checked for possible relevant studies. When multiple publications of the same study appeared, only the most recent and/or most complete reporting study were included. We retrospectively collected clinical data of cancer patients samples from three melanoma studies^17–19^, two lung cancer trials^20–22^, one renal cancer datasets^23^, and two cohorts, including multiple tumors^24,25^. After removing patients samples without survival information, a total of 2734 patients treated with ICIs were included in this study.

### Statistics

Survival analysis was analyzed by Kaplan–Meier method and compared using log-rank test. It was censored at the last date that the patient was not dead. HR was calculated by Cox proportional hazards model and 95% CI was reported. Median OS time and 95% CI were presented where relevant. Spearman’s *ρ* correlation coefficient was calculated. The relations between various clinical characteristics and *SETD2* mutation were evaluated with *χ*^2^ test, Student’s *t* test, or Fisher’s exact test depending on the context. Two-sided *P* < 0.05 was considered statistically significant. All statistical analysis was conducted by MedCalc 18.2.1 (MedCalc Software, Belgium).

### Reporting summary

Further information on research design is available in the Nature Research Reporting Summary linked to this article.

## Supplementary information

Supplementary Information

Reporting Summary

## Data Availability

The datasets generated during and/or analyzed during the current study are available from the corresponding author upon reasonable request.

## Code availability

The code that supports the findings of this study are available from the corresponding author upon reasonable request.
