# Supplementary material for: Pan-cancer analysis of SETD2 mutation and its association with the efficacy of immunotherapy
Source: NPJ Precis Oncol. 2021 Jun 14;5:51. doi: 10.1038/s41698-021-00193-0 (PMC8203790; doi:10.1038/s41698-021-00193-0)
Supplement: Supplementary file 1 — Supplementary Information [file 41698_2021_193_MOESM1_ESM.docx]

**Supplemental Figure 1** Tumor mutation burden (a) and MSIsensor score (b) in various tumor histologies stratified by *SETD2* mutation status. *, *P*<0.05; **, *P*<0.01; ***, *P*<0.001.


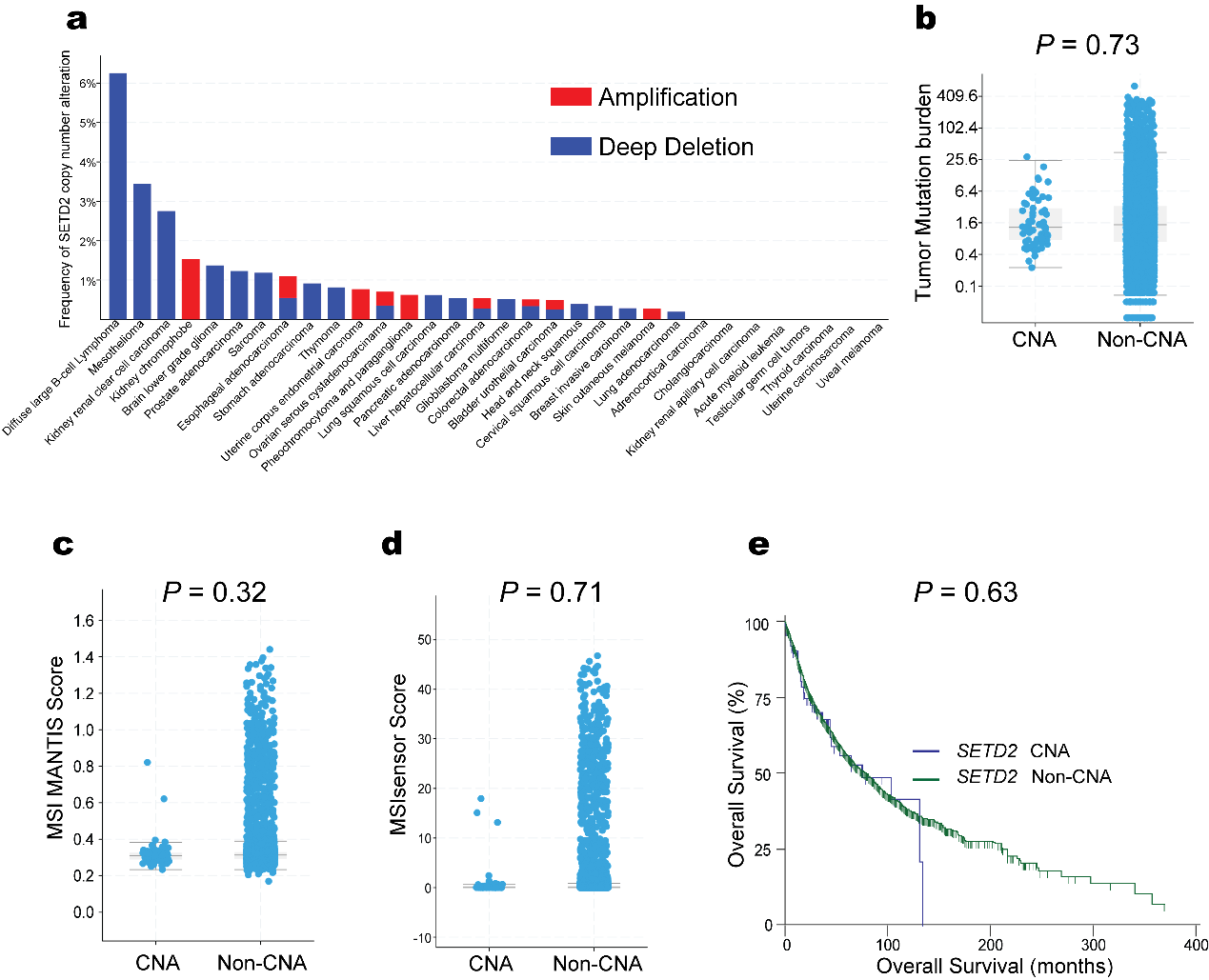


**Supplemental Figure 2** The characteristics of *SETD2* copy number alteration (CNA) in TCGA pan-cancer cohort.

1. The prevalence of *SETD2* CNA across tumors.
2. Tumor mutation burdens in *SETD2* non-CNA samples and *SETD2* CNA samples.
3. MSI MANTIS scores in *SETD2* non-CNA cancer and *SETD2* CNA cancer.
4. MSIsensor scores in *SETD2* non-CNA cancer and *SETD2* CNA cancer.
5. Kaplan-Meier survival analysis stratified by *SETD2* CNA status.
